# Glucose-6-phosphatase deficiency

**DOI:** 10.1186/1750-1172-6-27

**Published:** 2011-05-20

**Authors:** Roseline Froissart, Monique Piraud, Alix Mollet Boudjemline, Christine Vianey-Saban, François Petit, Aurélie Hubert-Buron, Pascale Trioche Eberschweiler, Vincent Gajdos, Philippe Labrune

**Affiliations:** 1 Laboratoire des Maladies Héréditaires du Métabolisme et Dépistage Néonatal, Centre de Biologie et de Pathologie Est, Hospices Civils de Lyon, 59 Boulevard Pinel, 69677 Bron cedex, France; 2 INSERM U820, Université Claude Bernard, 69008 Lyon, France; 3 Centre de Référence Maladies Héréditaires du Métabolisme Hépatique, Service de Pédiatrie, APHP, Hôpital Antoine Béclère, 157 rue de la porte de Trivaux, 92141 Clamart cedex, Université Paris Sud, UFR Kremlin Bicêtre, 63 rue Gabriel Peri, 94276 Le Kremlin Bicêtre cedex, France; 4 INSERM U 948, CHU Nantes, 44020 Nantes cedex, France

## Abstract

Glucose-6-phosphatase deficiency (G6P deficiency), or glycogen storage disease type I (GSDI), is a group of inherited metabolic diseases, including types Ia and Ib, characterized by poor tolerance to fasting, growth retardation and hepatomegaly resulting from accumulation of glycogen and fat in the liver. Prevalence is unknown and annual incidence is around 1/100,000 births. GSDIa is the more frequent type, representing about 80% of GSDI patients. The disease commonly manifests, between the ages of 3 to 4 months by symptoms of hypoglycemia (tremors, seizures, cyanosis, apnea). Patients have poor tolerance to fasting, marked hepatomegaly, growth retardation (small stature and delayed puberty), generally improved by an appropriate diet, osteopenia and sometimes osteoporosis, full-cheeked round face, enlarged kydneys and platelet dysfunctions leading to frequent epistaxis. In addition, in GSDIb, neutropenia and neutrophil dysfunction are responsible for tendency towards infections, relapsing aphtous gingivostomatitis, and inflammatory bowel disease. Late complications are hepatic (adenomas with rare but possible transformation into hepatocarcinoma) and renal (glomerular hyperfiltration leading to proteinuria and sometimes to renal insufficiency). GSDI is caused by a dysfunction in the G6P system, a key step in the regulation of glycemia. The deficit concerns the catalytic subunit G6P-alpha (type Ia) which is restricted to expression in the liver, kidney and intestine, or the ubiquitously expressed G6P transporter (type Ib). Mutations in the genes *G6PC *(17q21) and *SLC37A4 *(11q23) respectively cause GSDIa and Ib. Many mutations have been identified in both genes,. Transmission is autosomal recessive. Diagnosis is based on clinical presentation, on abnormal basal values and absence of hyperglycemic response to glucagon. It can be confirmed by demonstrating a deficient activity of a G6P system component in a liver biopsy. To date, the diagnosis is most commonly confirmed by *G6PC *(GSDIa) or *SLC37A4 *(GSDIb) gene analysis, and the indications of liver biopsy to measure G6P activity are getting rarer and rarer. Differential diagnoses include the other GSDs, in particular type III (see this term). However, in GSDIII, glycemia and lactacidemia are high after a meal and low after a fast period (often with a later occurrence than that of type I). Primary liver tumors and Pepper syndrome (hepatic metastases of neuroblastoma) may be evoked but are easily ruled out through clinical and ultrasound data. Antenatal diagnosis is possible through molecular analysis of amniocytes or chorionic villous cells. Pre-implantatory genetic diagnosis may also be discussed. Genetic counseling should be offered to patients and their families. The dietary treatment aims at avoiding hypoglycemia (frequent meals, nocturnal enteral feeding through a nasogastric tube, and later oral addition of uncooked starch) and acidosis (restricted fructose and galactose intake). Liver transplantation, performed on the basis of poor metabolic control and/or hepatocarcinoma, corrects hypoglycemia, but renal involvement may continue to progress and neutropenia is not always corrected in type Ib. Kidney transplantation can be performed in case of severe renal insufficiency. Combined liver-kidney grafts have been performed in a few cases. Prognosis is usually good: late hepatic and renal complications may occur, however, with adapted management, patients have almost normal life span.

**Disease name and synonyms:**

Glucose-6-phosphatase deficiency or G6P deficiency or glycogen storage disease type I or GSDI or type I glycogenosis or Von Gierke disease or Hepatorenal glycogenosis.

## Definition and diagnostic criteria

Glycogen storage disease type I (GSDI) is a group of rare inherited diseases resulting from a defect in the glucose-6-phosphatase (G6Pase) system which has a key role in glucose homeostasis as it is required for the hydrolysis of glucose-6-phosphate (G6P) into glucose and inorganic phosphate (Pi). The main diagnostic criteria are: hepatomegaly, fast-induced hypoglycemia with hyperlactacidemia, and hyperlipidemia. Two main subtypes are unambiguously recognized: GSD type Ia (GSDIa) due to a defect of the catalytic unit G6Pase-alpha (or G6PC), and GSD type Ib (GSDIb) due to a defect of the glucose-6-phosphate translocase (or G6PT) [1,2]. The existence of other types (type Ic and type Id) has not been confirmed [3,4].

## Epidemiology

GSDI has an estimated annual incidence of around 1/100,000 births, representing approximately 30% of hepatic GSD and with GSDIa being the most frequent type (about 80% of the GSDI patients)[1]. GSDIa is particularly common in the Ashkenazi Jewish population, in which the carrier frequency for the p.R83C allele was found to be 1.4%, predicting a prevalence five times higher than in the general Caucasian population [5].

## Clinical description [1,2,6]

***GSDI patients ***may present with fast-induced hypoglycemia (sometimes occurring rapidly in about 2 to 2 and a half hours after a meal) and hyperlactacidemia in the neonatal period. More commonly, the first symptom is the presence of a protruded abdomen due to marked hepatomegaly around 3 months of age, though in some cases the liver may already be enlarged at birth. The liver size gradually increases and the lower border may reach below the umbilicus. It must be stressed that the hepatomegaly may be missed on physical examination, as the liver is soft. Hepatocellular adenomas are usually asymptomatic and physical examination is rarely contributive, except in very rare cases when adenomas are superficial. Fasting tolerance is very limited: hypoglycemia, which may cause convulsions, and lactic acidemia, account for the initial gravity of the disease. In some cases, the hypoglycemia may be less symptomatic since lactate may be used as a cerebral metabolic fuel. The other biological hallmarks are hyperlipemia and hyperuricemia. The full-cheeked, round "doll like" face, and a protruding abdomen contrast with the thin limbs. Growth delay and late onset of puberty [2,7] are very frequent signs, which can be improved by good metabolic control [8]. Osteopenia [9] is commonly found and it has been suggested that the subclinical muscle weakness could also contribute to the low bone mass [10]. Chronic acidosis and hypertriglyceridemia also play an important role in the development of osteopenia. Kidneys are enlarged. Platelet dysfunction, which is related to dyslipidemia, explains the tendency for ecchymoses and bleeding. Anemia is commonly found. Intermittent diarrhea occurs in a number of patients. Ovarian cysts have also been reported. Recently, an increased prevalence of hypothyroidism (GSDIa and GSDIb) and thyroid autoimmunity (GSDIb) has been reported [11].

***Long-term complications ***[12-14] can be delayed by good metabolic control.

The development of hepatocellular adenomas (HCA) is a well-known complication. They are usually detected between the second and the third decades of life, and their frequencies range from 16 to 75% and are equal in both sexes, [2]. The diagnosis is rarely based on physical examination, except when superficial adenomas develop, making the liver surface uneven [15-18]. Usually, liver ultrasound, CT scan or MRI allow the diagnosis and regular follow-up is thus necessary During the first ten years, liver ultrasound should be performed once a year. Since the age of 10, liver MRI may be proposed each year. Specific MRI patterns, related to diffuse fat repartition and sinusoid dilatation have been aasociated with HNF-1α - mutated adenomas and inflammatory adenomas, respectively [19]. Furthermore, chemical-shift MRI has been useful in discriminating increased liver echogenicity in GSDs [20]. MRI also allows the quantification of hepatic steatosis. Should hepatic lesions be detected, the follow-up must be intensified, and liver MRI should be proposed every 6 months, sometimes combined with ultrasound examinations if MRI is more difficult to organize. CT scan may be useful, should a surgical resection of adenomas be plannned.

*Renal complications *start with silent glomerular hyperfiltration before the development of microalbuminuria then proteinuria, which can lead to renal failure [2]. Hypercalciuria is a consequence of renal distal tubular dysfunction and may contribute to renal calculi and/or nephrocalcinosis and osteopenia. Hypocitraturia has been reported as a risk factor for nephrocalcinosis [21,22]. Oxidative stress has also been reported as a mechanism underlying GSD-Ia nephropathy [23]. When microalbuminuria has been detected and confirmed, treatment with angiotensin converting enzyme inhibitors must be started without delay [24]

*Hyperuricemia *[25] must be treated because it can lead to gout and, above all, it participates (together with hypocitraturia) in the constitution of stones, nephrocalcinosis and renal failure [2].

*Severe hypertriglyceridemia *seems to increase the risk of pancreatitis [26] but, to date, the risks of atherosclerosis and early cardiovascular complications do not seem to be increased [2,14,27-29]. However, a recent study of 28 patients with GSDI (mean age 23 years) reported arterial dysfunction which was characterized by increased carotid intima media thickness and a higher augmentation index measured by radial artery tonometry [30].

*Pulmonary hypertension *is a very rare complication and its prognosis is very poor [15,31,32]. Its physiopathology is not well known; abnormalities in the platelet metabolism of serotonin have been suspected [32].

### GSDIb patients

In addition to these signs that characterize GSDIa, GSDIb patients generally present with neutropenia that appears not to be due to a defect in bone marrow production. Neutrophils and/or monocytes are functionally abnormal, showing decreases in respiratory burst and motility in response to stimuli [3,4]. This dysfunction is responsible for recurrent infections, oral and intestinal mucosal ulcerations and inflammatory intestinal diseases suggestive of Crohn's disease [33-35]. This condition has occurred in over 77% of patients with GSDIb by adulthood [33]. The development of diarrhea, persistent abdominal pain, unexplained fever, gastrointestinal bleeding, perianal lesions should lead to further evaluation. Colonoscopy must be performed; should it be negative, endoscopic evaluation of the small bowel has to be considered. [36]. Recently, elevated levels of anti-bacterial flagellin antibodies (anti-CBir1) have been detected in the serum of 17/19 GSDIb patients [37]. As these antibodies have been associated with Crohn disease in the general population, these data are interesting. However, in this study, the antibody did not discriminate patients with and without inflammatory bowel disease and long-term follow-up is necessary to know whether these antibodies can predict the occurrence of intestinal inflammation [37].

A few of the reported patients (about 10%) did not present with neutropenia and/or neutrophil dysfunction [38-40]. Interestingly, one of these patients [38] had a mutation that only partially affected the activity of the transporter [41]. In contrast, neutropenia is very rare in GSDIa patients [42]. Splenomegaly, which is very rarely found in GSDIa patients, was reported (together with hepatomegaly) more frequently in GSDIb patients [2,43], specially in those receiving treatments with G-CSF.

### Pregnancy

Fertility is normal in GSDI patients and several pregnancies have been reported in affected women. Close monitoring of these pregnancies is required due to the risk of exacerbating the renal problems, of the enhanced danger of hemorrhages and of the need to provide satisfying metabolic control for the fetus [44]. In GSDIa mothers, most neonates have been delivered by cesarean section [45]. In GSDIb mothers, five successful pregnancies in three patients have been recently reported [46]. There were no major complications related to neutropenia except for oral ulcers and all neonates were delivered vaginally.

Developping a strategy for an optimal management of contraception in GSD I women is crucial. Ethynyloestradiol should be avoided because of a link with hepatic adenomas and is contraindicated in patients with hypertriglyceridemia and hypercholesterolemia. Blockage of ovulation can be achieved using high doses of progestogen alone, administered from the 5^th ^to the 25^th ^day of the cycle. Another possibility is based on daily administrations of low doses of progestogen [44]. Mechanical contraception using intra-uterine device is controversial in such patients.

## Etiology

### Enzyme deficit

GSDI was first described by von Gierke in 1929. In 1952, Cori and Cori showed that the disease (called GSDIa) was caused by a deficit in G6Pase, an enzyme expressed mainly in the liver and kidney and, to a lesser degree, in the intestine. Subsequently, it was found that some patients are not deficient in G6Pase, even though a number of functional tests demonstrated their inability to degrade G6P *in vivo*: this condition was called GSDIb. To explain this defect, Arion *et al *[47] hypothesized that G6P hydrolysis required the participation of several proteins located in the endoplasmic reticulum (ER) membrane: a catalytic unit (G6Pase) capable of hydrolyzing several phosphate esters (G6P, mannose-6-phosphate, carbamylphosphate and pyrophosphate) and a G6P-specific bidirectional translocase (G6PT), which would assure its entry into the lumen of the endoplasmic reticulum, where G6Pase exerts its action. Unlike G6Pase, G6PT is expressed ubiquitously. G6Pase and G6PT were found to be co-dependent as G6Pase activity is required for efficient transport of G6P into the ER lumen [48].

On the basis of kinetic studies, Arion *et al. *[47] proposed in 1980 a multicomponent model with specific transmembrane transporter proteins for G6P (T1), phosphate, pyrophosphate and carbamylphosphate (T2) and glucose (T3). Nordlie *et al *[49] described a patient with GSDIc who had a deficit in the bidirectional translocase (T2) which allows phosphate resulting from G6P hydrolysis to leave the endoplasmic reticulum. The existence of GSD type Ic was discussed when mutations in the gene encoding G6PT were identified in most of these patients [50]. However, no mutation was found in the gene encoding G6PT in the patient originally described by Nordlie [50,51], thereby suggesting that another protein could be involved in this patient or that the patient was affected by a different disorder. The hypothesis that the gene coding for a microsomal phosphate transporter (NPT4) was mutated in GSDIc patients devoid of mutations in the G6PT gene could not be confirmed [52]. Recently, it has been demonstrated that G6PT is an organophosphate:Pi antiporter transporting G6P into the ER lumen and Pi out. This supports that GSDIb and GSDIc which are deficient in the same G6PT gene represent a single disease [53].

The existence of GSD type Id, attributed to a deficit in translocase (T3) has never been proven and the hypothesis that the glucose transporter GLUT7 could be involved was ruled out when mutations were identified in the gene encoding G6PT [50].

The G6Pase deficient in GSDIa was subsequently renamed G6Pase-alpha as another G6P hydrolase named G6Pase-beta (or G6PC3) has been identified. G6Pase-beta is ubiquitously expressed and can form a complex with G6PT in non gluconeogenic organs, which could explain why endogenous glucose is still produced in GSDIa patients [6,54]. However, Wang et al [55] showed that the deletion of the gene encoding the G6Pase-beta in mice does not result in hypoglycemia and that the phenotype of knock-out mice is mild.

The role of G6PT, in addition to that in the glucose-6-phosphatase system, has not yet been fully elucidated. A role in the differentiation of neutrophils has been suggested [56] and studies in mice model have shown that G6PT is also an important immunomodulatory protein [57]. Recent studies provided evidence that neutrophil homeostasis and function is linked to the endogenous glucose production via the G6PT/G6Pase-beta complex. G6PT deficient or G6Pase-beta deficient neutrophils are unable to produce endogenous glucose and would manifest enhanced ER stress and apoptosis, which could contribute to neutrophil dysfunction in GSDIb patients [58]. Cheung et al [59] showed that mice lacking the G6Pase-beta had impaired neutrophil activity and increased susceptibility to bacterial infection. G6Pase-beta deficiency has been recently identified in patients presenting a severe congenital neutropenia syndrome [60].

### Metabolic alterations

In GSDI, the fasting hypoglycemia results from the blockage of the last step of glycogenolysis and gluconeogenesis. However, a few patients show an unexpected tolerance to fasting [61].

Hyperlactacidemia and glycogen storage are due to excess G6P which cannot be metabolized to glucose. Thus, galactose, fructose and glycerol will not correct hypoglycemia and will contribute to hyperlactacidemia. Hyperlipidemia is a result of both increased synthesis from excess of acetyl-coenzyme A *via *malonyl-coenzyme A (the first step of fatty acid synthesis), and decreased lipid serum clearance, though mechanisms of both hyperlipidemia and liver steatosis are not completely understood [62]. Accumulation of fats in the liver significantly contributes to the hepatomegaly.

Increased malonyl-coenzyme A inhibits carnitine palmytoyltransferase I, resulting in reduced ketone production and increased dicarboxylic aciduria. Hyperuricemia [1,6] is caused by both decreased renal clearance (lactate competes with uric acid) and increased synthesis (decreased intrahepatic phosphate concentration stimulates the degradation pathway of adenine nucleotides).

### Mechanisms underlying the development of hepatocellular adenomas

The mechanisms underlying the development of HCAs are still not completely elucidated. However, a new classification of HCA and a better knowledge of their relationship with hepatocellular carcinoma was proposed by Zucman Rossi et al [63]. A recent study has reported chromosomal and genetic alterations in HCAs associated with GSDIa: simultaneous gain of 6p and loss of 6q, association of larger HCAs with chromosome 6 aberrations, reduced expression of IGF2R and LATS1 (candidate tumour suppressor genes) at 6q in more than 50% of HCAs associated with GSDIa [64]. The activation of β-catenin has been reported to be an important risk factor for malignant degeneration [63]. To date, the presence of activated β-catenin has been reported in 3 GSDIa HCAs, among which one was associated with a carcinoma.

The role of metabolic control in adenomas formation has also been discussed. In a case-control retrospective study, no significant differences in metabolic balance could be detected between GSDI patients who developed adenomas and those who did not [65].

Another mechanism could involve serum cytokines. Kim et al [66] have reported that GSDIa mice exhibit a persistent increase in peripheral blood neutrophil counts along with elevated levels of granulocyte colony stimulating factor (G-CSF) and cytokine-induced neutrophil chemoattractant. The authors suggest that there is a progressive low level of immune challenge since there is a long-term damage to the liver in GSDI patients despite dietary controls [66].

### Refractory iron deficiency anemia

Severe refractory iron deficiency anemia has been reported in GSDIa patients with hepatocellular adenomas, that is alleviated with either adenoma resection or liver transplantation [67,68]. The role of hepcidin, a propeptide that is converted in three mature peptides and which limit both release of iron from macrophages and intestinal iron absorption, has been proposed [68]. High levels of hepcidin mRNA expression were found in the adenomas of anemic GSDIa patients whereas hepcidin mRNA expression was decreased in the unaffected liver [68]. The upregulated production of hepcidin in adenomas would result in the dysregulation of the iron utilization cycle in GSDIa patients [68]. More studies are required to continue to shed some light on the precise mechanisms of anemia in such patients.

### Mutations and genotype/phenotype correlations

#### GSDIa

Human G6Pase gene *(G6PC) *was isolated by Lei *et al. *[69]. The gene has been localized to chromosome 17 at 17q21, spans 12.5 kb, includes 5 exons and codes for a highly hydrophobic protein of 357 amino acids containing 9 transmembrane helixes. Its promoter contains several response elements for glucocorticoids, cyclic AMP and insulin, and is regulated by hepatocyte nuclear factors that control its expression [70].

Over 550 unrelated patients affected with GSDIa have been studied worldwide and more than 85 mutations (Human Gene Mutation Database; http://www.hgmd.cf.ac.uk, [71]) have been identified. The majority are missense mutations (64%) [38,41,72-74] and all of them are small gene alterations. A few alleles could not be identified. Only some of the mutations have a significant frequency [75]:

- in the Caucasian population, p.R83C and p.Q347X are found in 33% and 18% of the GSD Ia alleles, respectively.

- in Jewish patients, p.R83C is particularly frequent (98% of the alleles) and p.Q347X is found in the remaining alleles (2%).

- in Hispanic Americans, the c.380_381insTA mutation represents about half of the alleles.

- in Japanese patients, the p.Q347X and p.R83C mutations have never been found, p.R83H is very rare, but the silent nucleotide change c.648G > T, responsible for the deletion of 91 nucleotides in exon 5 in the cDNA, is present in 91% of the GSDIa alleles in this population.

- in Chinese patients, c.648G > T is also frequent (54% of the alleles). The p.Q347X mutation has never been found and p.R83C only once, while p.R83H is present in 26% of the mutated alleles.

- in Korean patients, c.648G > T is also the most frequent mutation (75%).

- in Tunisian patients, p.R83C and p.R170Q accounts for 67% and 28% of the alleles respectively [76].

The structure and function of fifty missense mutations and the in-frame deletion **p**.F327del have been studied [73,77]. The 5 active site mutations (including the p.R83C/H mutations), 23 of the 32 helical mutations and 8 of the 14 non helical mutations abolish completely the activity (altering protein stability and/or catalytic capacity). The remaining studied missense mutations are associated with some residual activity. The results of this study [77] should help facilitating genotype-phenotype delineation, at least for patients homozygous for a studied mutation. A Japanese patient homozygous for the non helical p.P257L mutation had a very mild phenotype, in accordance with expression study, and experienced no hypoglycemic episodes. The clinical phenotype of patients carrying these "mild" mutations should be precisely documented in order to try to determine the minimal G6Pase activity required for avoiding hypoglycemic episodes. The c.648G > T homozygous status was associated with a milder phenotype with respect to hypoglycemic events [42] but other genetic and/or acquired factors may have influence on a possibly increased risk for hepatocellular carcinoma [78]. Interestingly, it was found that p.G188R homozygosity confers an atypical GSDIb phenotype with neutropenia and recurrent infections [79].

#### GSDIb

The human G6PT gene (*SLC37A4*) has been localized to chromosome 11 at 11q23 [80], spans 4.5 kb, contains 9 exons and codes for a highly hydrophobic protein containing 10 transmembrane domains [81,82]. Unlike G6Pase, G6PT is expressed in many tissues and tissue-specific splicing is responsible for several variants, the significance of which has not yet been elucidated [82]. It is highly expressed in the liver, pancreas, kidney and hematopoietic progenitor cells [54]. In the liver and leucocytes, exon 7 is not expressed. *SLC37A4 *gene expression is regulated, like that of *G6PC*, by glucose, insulin and cyclic AMP [70].

Over 160 patients have been studied worldwide to date [38,42,49,80-82] and more than 81 mutations have been identified (Human Gene Mutation Database; http://www.hgmd.cf.ac.uk, [71]).

All are small size gene alterations except one case of large deletion [83]. Most of them are missense mutations (40%). No *SLC37A4 *gene mutations have been found in exon 7, the 5' part of exon 1 and the 3' portion of exon 9, which are not expressed in the liver transcript. Molecular heterogeneity is extreme but several mutations predominate and vary according to the population: c.1042_1043delCT and p.G339C are the most common in the Caucasian population, where they represent nearly half of the G6PT alleles, and the p.W118R mutation is present in nearly 40% of the Japanese G6PT alleles [42].

A functional assay for G6P transport has been developed and used to characterize 30 of the mutations: 20 of them completely abolish G6P uptake activity, while 10 retained residual activity including the p.G339C mutation [84]. Additionally, a three dimensional structural model was built by homology modelling in order to predict *in silico *the effect of mutations [85]. No correlation was found between individual mutations and the absence of neutropenia, bacterial infections and systemic complications [30,86].

## Diagnosis

### Biochemical methods of diagnosis

*Biological hallmarks: *fasting tolerance is poor and fasting blood analyses reveal hypoglycemia, hyperlactacidemia, hypertriglyceridemia, hypercholesterolemia and, in many cases, hyperuricemia.

*Functional tests *show the absence of a glycemic response and an aggravation of hyperlactacidemia after injection of glucagon (1 mg/m^2 ^of body surface) in a fasting patient or 2 hours after a meal rich in carbohydrates. Galactose injection (1 g/kg) neither induces hyperglycemia nor corrects the hypoglycemia.

*Other biological abnormalities *include hypoacetonemia

hypoinsulinemia and hyperglucagonemia. Additionally, marked elevation of serum biotinidase activity has been reported in GSDIa patients [87].

*Study of the G6Pase system in a liver biopsy *[88]

The biochemical diagnosis of GSDI requires a liver biopsy (ideally fresh, not frozen), sufficiently large to enable the analysis of the different constituents of the G6Pase system. A homogenate is prepared under conditions maintaining or not microsomal membrane integrity. Hydrolytic activity is measured using several substrates: mannose-6-phosphate (to evaluate microsomal membrane integrity), G6P and pyrophosphate. In type Ia, hydrolytic activity is defective, regardless of the substrate used and the status of microsomal membranes. In type Ib, G6P hydrolysis is defective when microsomal membranes are intact [89].

No biochemical phenotype-clinical phenotype correlations could be established based on the results of residual activity and glycogen storage in the liver.

### Histopathological findings

Histology of the liver shows glycogen and fat-induced hepatocytic distension. Fibrosis may also be present in some patients.

### Molecular studies

Complete sequencing of the *G6PC *(GSDIa) and *SLC374A *(GSDIb) genes allows diagnosis in nearly all patients with evocative clinical and biochemical signs of GSDI, thereby eliminating the need for a liver biopsy.

## Differential diagnosis

Nearly all cases are diagnosed in infancy. A limited number of clinical and biological parameters allow the diagnosis: marked and soft hepatomegaly associated with hypoglycemic episodes and elevated fasting blood lactate concentration decreasing after a carbohydrate rich meal or a glucose tolerance test are indicative of GSDI. Diagnosis may be more difficult in older patients, especially in the few patients who present an unusual prolonged tolerance to fasting [6,90].

Among other gluconeogenesis disorders, fructose-1, 6 diphosphatase deficiency can be discussed in some cases. However, in this disease, the tolerance to fasting is much longer (8 to 10 hours) than it is in GSDI, and the liver is not as enlarged.

Other types of GSD may be evoked. GSDIII (amylo-1-6-glucosidase deficiency) may be clinically similar in infancy but hypoglycemia is usually not as severe as in type I because gluconeogenesis is intact and phosphorylase is still able to metabolize peripheral branches of glycogen. In GSDIII, lactatemia at fast and uric acid are typically normal, and liver transaminases levels are higher than in GSDI. GSD type VI (hepatic phosphorylase deficiency) or type IX (hepatic phosphorylase b kinase deficiency) should be also evoked as a severe presentation of these diseases may exist.

Primary liver tumors and Pepper syndrome (hepatic metastases of neuroblastoma) may be evoked but are easily ruled out through clinical and ultrasound data.

## Genetic counseling

The disease is transmitted as an autosomal recessive trait. Both parents of an affected child are heterozygotes. The risk of recurrence is 25%, at each pregnancy. Identification of both mutations in the patient enables the diagnosis of potential heterozygotes in the family.

## Prenatal diagnosis

The good response of most patients to dietary control has rendered prenatal testing relatively rare. Nonetheless, some children respond poorly to the diet and will require a liver transplant. In addition, GSDIb is often accompanied by severe infections and inflammatory bowel disease.

Before the discovery of the gene, prenatal diagnosis of GSDIa required a fetal liver biopsy obtained late in pregnancy, and was subject to diagnostic errors. Prenatal diagnosis of GSDIb had never been achieved.

To date, molecular studies have made prenatal diagnosis of GSDIa and GSDIb easy to perform when the familial mutations have been identified [91-93]. Fetal DNA may be extracted from chorionic villi or from amniotic fluid cells.

## Management including treatment [1,2,6,30,94]

Detailed guidelines for patient's management based on the data of the European Study on Glycogen Storage Disease type I have been provided [95].

### Dietary treatment

Treatment aims at preventing hypoglycemia in order to avoid neurological involvement and long-term complications (hepatic, renal, *etc.*) and to assure normal growth.

Treatment is essentially dietary and consists of frequent meals, continuous nocturnal nasogastric drip feeding, ingestion of slow-absorption carbohydrates (uncooked starch: [96]), and restricted intakes of both fructose and galactose which can aggravate hyperlactacidemia.

Daily caloric intake must be monitored: insufficient intake does not correct the metabolic disorder (hypoglycemia, hyperlactacidemia and hyperuricemia) and leads to retarded growth, whereas excessive intake increases the glycogen overload, hepatomegaly and hyperlipidemia, and causes obesity. The diet must provide 60-65% of total caloric intake from carbohydrates, 10-15% from proteins and the reminder from fat.

*Until 12 months, *frequent meals (5 meals per day) and continuous nocturnal feeding *via *a nasogastric tube, providing 6-8 mg of glucose/kg/min, are recommended. However, continuous nocturnal feeding may be, in some cases replaced by nocturnal meals.

*After 1 year*, uncooked cornstarch (which may be introduced from 9 to 12 months of age, with progressive increase of doses) can, in some patients, replace the continuous nocturnal feeding at the initial dose of 0.5 g/kg then slowly increased to 1 g/kg every 4 hours. As the child grows older, the cornstarch regimen can be changed to the dose of 1.5-2.0 g/kg every 6 hours.

Another therapeutic regimen may be proposed and discussed for cornstarch which can be started during infancy, and given between meals or before bed so as not to interfere with appetite at meal time (94). Recommendations for dosing are: 1.6 g/kg body weight every 4 hours for infants, 1.7-2.5 g/kg body weight every 6 hours for young children through puberty, and 1.7-2.5 g/kg body weight given before bed time for adults. A novel and modified starch is being tested in GSD patients. To date, it seems that this new starch might allow, in some patients, longer duration of euglycemia and better short-term metabolic control [97,98]. This starch is allowed in several countries (United Kingdom, Netherlands, Germany).

*In adults*, the cornstarch dose and the intake interval can be increased. However, several adults stop taking cornstarch, even though they are encouraged to continue.

Treatment efficacy is evaluated by monitoring clinical (growth curve, body mass index, importance of hepatomegaly, blood pressure) and biological parameters. The preprandial glycemia must be above 3.5 mmol/L and adjusted to the actual urinary lactate excretion, which must be below 0.06 mmol/mmol creatinine in 12 hours urine samples (night and day). Blood lactate monitoring, when available, may be useful for supplementing glucose monitoring [99]. Hyperlactatemia causes acute clinical deterioration whereas chronic hyperlactatemia has been associated with long-term complications of GSDI. The main use of lactate monitoring is during intercurrent illness, when the rapid development of lactic acidosis is very likely. In such situations, the use of a portable lactate meter seems to be a valuable tool [99]. Triglyceridemia, cholesterolemia, uricemia, blood gazes, proteinuria and complete blood cell count should be measured at each outpatient visit.

Portocaval shunts were recommended in the past but accorded very limited clinical benefit and have been abandoned, since 1984 [4].

Surgery requires special care [4] in these patients at increased risk of hemorrhages and metabolic imbalances (hypoglycemia and hyperlactacidemia): glycemia must be maintained (perfusions of 10% glucose before, during and after the intervention) and solutions containing lactate should be avoided (Ringer's, for example). Corticosteroids may be discussed for a short period even though their use is usually contraindicated., and careful follow-up and correction of hemostasis abnormalities are mandatory.

***Therapeutic adjuvants ***include vitamin supplements (vitamins D and B1, *etc.*), calcium (considering limited milk intake), iron in case of anemia (after excluding other causes) and allopurinol when hyperuricemia is present. If permanent microalbuminuria is present, treatment with angiotensin-converting enzyme (ACE) inhibitor is started with the aim of preventing renal complications [24], and additional blood pressure lowering drugs may be added if blood pressure remains elevated. Should the patient become pregnant, ACE inhibitors must be stopped at once. Hyperlipemia only responds partially to dietary treatment, but triglyceride lowering drugs are not indicated if the level remains below 10 mmol/L. Cholesterol lowering drugs are not indicated in young patients because of the low risk of atherogenicity.

***For GSD type Ib***, granulocyte colony-stimulating factor (G-CSF) is able to correct the neutropenia, reduces the severity of bacterial infections and attenuates inflammatory intestinal disease [33,100]. Chronic G-CSF therapy may consist in two to three weekly injections, the average dose per injection being about 5 μg/kg body weight. G-CSF therapy must be carefully monitored and untoward effects may develop such as splenomegaly, thrombocytopenia, renal carcinoma [101]. Should pegylated G-CSF be used, other side effects must be known such as the occurrence of Sweet syndrome in one patient [102], respiratory distress and sudden death in another patient [101]. Careful monitoring of the patient's spleen size, total blood cell counts and bone density is recommended in GSD Ib patients receiving G-CSF [35,100].

Colitis is often treated with success using a combination of G-CSF and 5-aminosalicylic acid derivatives [36,100]. However, in a few cases, this treatment fails and other therapeutic approaches must be discussed. Corticosteroids are generally avoided in such situations, owing to steroid-induced glycogenolysis and the possibility of lactic acidosis and hyperlipidemia., Immunosuppressive drugs such as methotrexate, azathioprine and 6-mercaptopurine carry the risk of excessive immunosuppression and worsening neutropenia in GSD Ib patients. Adalimumab, a fully humanized monoclonal anti-TNF has been reported to be successful in one GS Ib patient with inflammatory bowel disease who was refractory to usual medical treatment [36]. Fecal alpha-1 antitrypsin must be monitored for evaluation of inflammatory bowel disease in GSDIb [38].

### Detection and follow-up of complications

Even though the risk of malignant transformation of adenomas into hepatocellular carcinomas appeared to be low [2,16,17], ie no malignant transformation had been reported in the European retrospective study, some recent reports suggested that the older the patients get, the more important the risk of transformation becomes [103]. The management of adenomas remains empirical; it may be expectant or surgical. Clinically, should abdominal pains occur and require major painkillers to be controlled, malignant transformation must be suspected, even though these manifestations are not specific. Biological markers (serum α fetoprotein and ACE) are not predictive of malignant transformation. Liver CT scan and MRI may be useful in such cases, even though further evaluations are needed. Surgical resection of hepatic adenomas is feasible. Previous reports have noted frequent haemorragic problems [104], but such complications may be avoided, provided meticulous metabolic control is obtained before surgery (personal data).

If dietary control fails or hepatic adenomas undergo malignant transformation, treatment of complications consists of liver transplantation [105]. Liver grafting corrects the hypoglycemia and the other biochemical anomalies but the correction of neutropenia is not constant [106,107]. It has not been proven that it can prevent renal involvement, which may even be worsened by the immunosuppressive therapy. However, there is limited experience of liver transplantation for GSDI given the rarity of the disease. Chronic allograft rejection, post-transfusion hepatitis C infection, renal failure, gouty arthritis, and portal vein thrombosis requiring re-transplantation have been reported [67]. The current literature shows that satisfactory medium-term outcomes can be achieved in GSD I patients (review in [67]). Similar data have been reported regarding living donor liver transplantation in such patients [108]. As pointed out by Davis and Weinstein (2008), the risks and benefits of liver transplantation should be carefully evaluated and the latter should only be performed when there risk for hepatocellular carcinoma or liver dysfunction is high [109]. Liver cell transplantation could provide a less invasive approach in the future [110]. It has been reported in a GSDIb patient with marked improvement up to 250 days following transplantation; however, long-term results are still unknown and the use of immunosuppressive agents is mandatory [111].

Bone marrow transplantation has been reported in one GSDIb patient who had life-threatening complications related to neutropenia and thrombocytopenia. It resulted in improved metabolic control and reduction of inflammatory bowel disease-related symptoms [112].

*Kidney *transplantation, performed in case of severe renal failure, does not correct hypoglycemia [103]. Should grafting be indicated, combined liver-kidney graft has been discussed when renal function is already compromised and successfully performed in a few cases [113,114].

## Prognosis [2,6,8]

Efficient and early dietary treatment has led to reduced mortality and morbidity, and most patients are able to live a fairly normal life. If normoglycemia is maintained, metabolic abnormalities and clinical parameters improve in most patients, though hyperlipidemia persists. The incidence of adenomas seems to be lower but renal disease cannot be completely avoided, even in good responders. Some patients do not respond well, continue to be short and may require liver or dual kidney/liver transplant (with the mortality and morbidity associated with these procedures). In GSDIb, good metabolic control may be more difficult to obtain because of recurrent serious infections and inflammatory bowel disease.

## Unresolved questions and ongoing research [3,4,17,115]

The nature of the interactions between G6Pase and G6PT, and the functions of G6PT other than in G6P transport, remain incompletely resolved. The mechanisms by which G6PT deficiency affects neutrophils functions and the role of endogenous glucose production via the G6PT/G6Pase-beta complex in normal neutrophil function remains to be elucidated. Other unsolved questions include the source of endogenous glucose production which increases with age leading to a better fasting tolerance, the etiology of most complications (growth retardation, liver adenomas, renal involvement, *etc.*) and the management of liver adenomas in the absence of clear-cut criterion to detect their early malignant transformation. Animal models should provide answers to these questions and should improve our understanding of the pathophysiology of these diseases.

The role of G6Pase in the small intestine in the control of glucose homeostasis has been well established [116]. Gluconeogenesis in the small intestine plays an important role in glucose balance. These data have been confirmed in the liver-specific G6Pase gene knock-out mice who do not exhibit hypoglycemia due to their intestinal (and renal) G6Pase activity [117]. In connection with this, more attention should be paid to intestinal manifestations in GSD I patients.

The efficacy of gene therapy has been studied in mouse and dog models. Adeno-associated virus (AAV) mediated therapy delivers the transgene to the liver of GSDIa mice, achieving long term correction [118]. In dogs, a significant correction of the GSDIa phenotype has been reported in two animals, using AAV mediated gene tranfer [119]. Only a few experiments were performed in the GSDIb model, but an improvement of metabolic profile and myeloid function was reported [120].

## Competing interests

The authors declare that they have no competing interests

## Authors' contributions

RF, MP and CVS wrote the biochemical and genetic part of the manuscript

FP and AH completed the molecular data and revised the biological part of the manuscript

PTE, AMB, VG and PL wrote the clinical part of the manuscript and revised the entire manuscript, specially after reviewing

All authors read and approved the final manuscript
